# Stress-Induced In Situ Modification of Transition Temperature in VO_2_ Films Capped by Chalcogenide

**DOI:** 10.3390/ma13235541

**Published:** 2020-12-04

**Authors:** Joe Sakai, Masashi Kuwahara, Kunio Okimura, Yoichi Uehara

**Affiliations:** 1 Institut Català de Nanociència i Nanotecnologia (ICN2), UAB Campus, ICN2 Building, 08193 Bellaterra, Spain; 2 National Institute of Advanced Industrial Science and Technology, Tsukuba-shi, Ibaraki 305-8560, Japan; kuwaco-kuwahara@aist.go.jp; 3 Graduate School of Science and Technology, Tokai University, Hiratsuka 259-1292, Japan; okifn@keyaki.cc.u-tokai.ac.jp; 4 Research Institute of Electrical Communication, Tohoku University, 2-1-1 Katahira, Aoba-ku, Sendai 980-8577, Japan; uehara@riec.tohoku.ac.jp

**Keywords:** vanadium oxide, chalcogenide, insulator-metal phase transition, phase change material, strain engineering

## Abstract

We attempted to modify the monoclinic–rutile structural phase transition temperature (*T*_tr_) of a VO_2_ thin film in situ through stress caused by amorphous–crystalline phase change of a chalcogenide layer on it. VO_2_ films on C- or R-plane Al_2_O_3_ substrates were capped by Ge_2_Sb_2_Te_5_ (GST) films by means of rf magnetron sputtering. *T*_tr_ of the VO_2_ layer was evaluated through temperature-controlled measurements of optical reflection intensity and electrical resistance. Crystallization of the GST capping layer was accompanied by a significant drop in *T*_tr_ of the VO_2_ layer underneath, either with or without a SiN*_x_* diffusion barrier layer between the two. The shift of *T*_tr_ was by ~30 °C for a GST/VO_2_ bilayered sample with thicknesses of 200/30 nm, and was by ~6 °C for a GST/SiN*_x_*/VO_2_ trilayered sample of 200/10/6 nm. The lowering of *T*_tr_ was most probably caused by the volume reduction in GST during the amorphous–crystalline phase change. The stress-induced in in situ modification of *T*_tr_ in VO_2_ films could pave the way for the application of nonvolatile changes of optical properties in optoelectronic devices.

## 1. Introduction

In optoelectronic components such as switches, waveguides, transistors, and memories, the operation principle requires the control of electronic signals by light irradiation or the control of photonic signals by an electric field. To realize such devices that are based on photon–electron interaction, materials that show phase transition accompanied by significant change in both electric properties (conductivity, etc.) and optical properties (reflectance, etc.) are strong candidates.

Vanadium dioxide (VO_2_) undergoes structural phase transition near room temperature (68 °C in a bulk under the atmospheric pressure) between a high-temperature phase with a rutile-type structure (R phase) and a low-temperature phase with a monoclinic structure (M phase) [1,2]. The electrical, optical, and thermal properties of VO_2_ abruptly change at the transition temperature (*T*_tr_). In the high-temperature R phase, reflectance in the infrared region and electrical conductivity significantly increase compared to the M phase. The M–R phase transition of VO_2_ can be induced not only by heat, but also by electric field [3,4], light [5,6], and mechanical strain [7], suggesting the possibility of realizing VO_2_-based electrical/optical switching devices operated with these stimuli.

When VO_2_ is in the M (or R) phase at a given temperature, its *T*_tr_ is supposed to be higher (lower) than that temperature. Therefore, if the *T*_tr_ can be tuned for a certain temperature range, it means that the phase can be switched reversibly between R and M in this temperature range. This stimulus-induced phase switching might lead one to expect that VO_2_ would be applied not only in optical switches, but also in electrical resistance change memory devices. However, the R phase induced by an external stimulus is volatile, i.e., it recovers to the M phase once the stimulus is removed. A certain continuous energy is generally required to maintain the R phase [8,9,10].

The use of strain could be a realistic approach to maintain the R phase of VO_2_, or to maintain the low *T*_tr_, without supplying continuous extrinsic energy. It is known that shortening of *c*_R_ axis is accompanied by lowering of *T*_tr_ in VO_2_. In what follows, the subscripts M and R indicate the phases of VO_2_. Muraoka and Hiroi reported the lowering of *T*_tr_ in VO_2_ films with short *c*_R_ axes grown on rutile TiO_2_ (001) substrates [11]. Cao et al. demonstrated a phase transition in a VO_2_ microbeam by applying mechanical stress, and revealed a wide-ranging relationship between *T*_tr_ and the strain along the *c*_R_ axis [7]. If the strain were nonvolatile and reversible, then so would be the modification of *T*_tr_, and bistability would be realized in a temperature range in which the *T*_tr_ could be modified by strain. Sources of strain in previous studies on the modulation of *T*_tr_ in VO_2_ films include lattice mismatches with the substrate or the underlayer [11,12,13] or doping using different elements [14,15]. However, the factor of modulation in these experiments was induced at the deposition stage, and hence, its *T*_tr_ was no longer controllable after the formation of the film.

Still, there are several ways to modulate the strain of a thin film in situ. Attempts have been made to control the strain of thin films using piezoelectric materials [16,17,18,19,20,21,22,23,24,25,26]. They include some studies on the modification of the magnetic properties of (La, Sr)MnO_3_ [16,17,18], CoFeB [19], and Ni [20] films, as well as some on the modification of the transition properties of VO_x_ films [22,23,24,25,26], all grown on piezo layers or piezo substrates. It has been reported that the *T*_tr_ of VO_2_ films can be controlled through the strain of (1−*x*)Pb(Mg_1/3_Nb_2/3_)–*x*PbTiO_3_ (PMN–PT) crystalline substrates by 1.35 °C [23] or by 6 °C [24]. Applying uniaxial pressure to a film with the tip of a scanning probe microscope also functions to induce local strain [27,28]. In the present report, we propose a method of capping the target thin film with a material in which amorphous–crystalline phase transition easily occurs. Generally, the density of a solid material in a crystalline phase is higher than that of the same composition material in an amorphous phase. Therefore, in a bilayered sample consisting of a strain-generator layer and VO_2_, one could expect that amorphous–crystalline phase changes in the strain-generator layer would cause in-plane compressive strain in the VO_2_ layer, resulting in the modulation of *T*_tr_ in VO_2_.

Ge_2_Sb_2_Te_5_ (GST) is a typical material that undergoes reversible and nonvolatile switching between amorphous and crystalline phases. A number of researchers have worked on the application of this material in the field of optical and electrical memory devices. Previously, we studied the optical and thermal properties of GST [29,30,31,32] and developed several optical devices using it [33,34,35,36,37]. The amorphous–crystalline phase change of GST is accompanied by a volume contraction of 6.8% [38]. Supposing an isotropic volume change, this value can be converted to a linear compressive strain by 1 − (1 − 0.068)^1/3^ = 2.3%. Assume that this strain is fully transferred to the VO_2_ layer in touch with the GST layer, and that the *c*_R_ axis of VO_2_ lies in-plane. According to the relationship between *T*_tr_ and the strain along *c*_R_ axis in VO_2_ microbeams, compressive strain for 2.3% along *c*_R_ could lower the *T*_tr_ by about 30 °C [7]. Moreover, previous reports on VO_2_ thin films grown on TiO_2_ (001) substrates implied that the strain effect on *T*_tr_ was more pronounced in thin films than in microbeams [11,39]. These reports showed *T*_tr_ values lower than those in the bulk by more than 50 °C in films with *c*_R_ compression of only −0.6%. The controllability of *T*_tr_ within several tens of °C implies switchability between the M and R phases in a range of several tens of °C, possibly satisfying the requirement of the operation temperature range of commercial devices.

To confirm the above concept, we herein report the in situ reduction of *T*_tr_ in a VO_2_ layer by amorphous–crystalline phase change in a GST layer that caps the VO_2_. During the preparation of this manuscript, we learned of a recent paper by Meng et al. on reflectivity measurements of GST/VO_2_ bilayered samples [40]. We would like to note that our study focuses on the modulation of *T*_tr_ in VO_2_, whereas they targeted the function as a four-value memory device by combining the phase changes of GST and VO_2_.

## 2. Experimental

### 2.1. Sample Preparation

**Figure 1:**
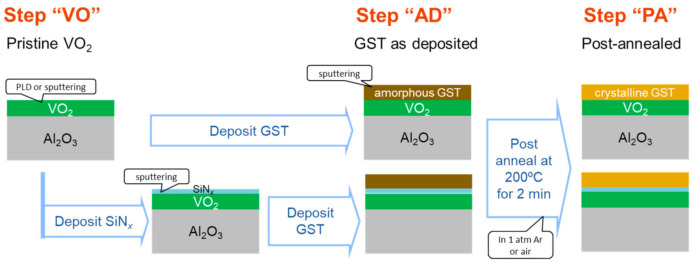
Preparation processes of GST/VO_2_ and GST/SiN*_x_*/VO_2_ multilayered samples.

Bilayered films consisting of VO_2_ and amorphous GST layers were prepared on sapphire substrates of either R-plane (1−102) or C-plane (0001). Figure 1 schematically shows the sample preparation processes. In order to realize the amorphous phase, the GST layer should be deposited after VO_2_, since the growth of crystalline VO_2_ films requires a temperature higher than the crystallization temperature of GST (161 °C) [41].

VO_2_ was grown by means of either pulsed laser deposition (PLD) or rf-biased reactive magnetron sputtering on substrates heated at 500 and 400 °C, respectively. A bias power of 5 W was applied to the sample stage during the sputtering process. Other deposition conditions can be found in previous reports [42,43].

**Figure 2:**
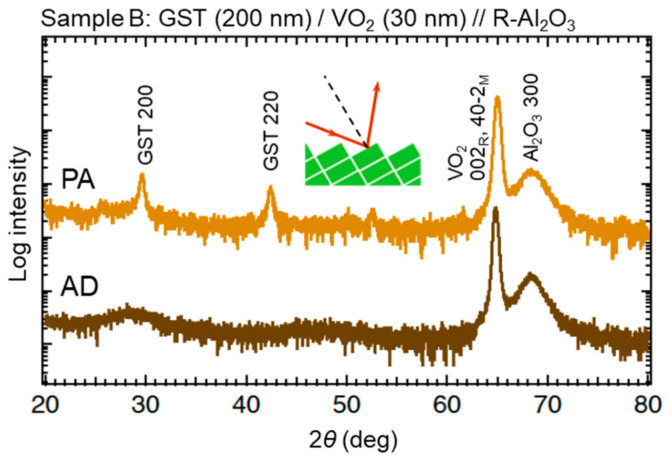
XRD 2*θ − ω* scan profiles of Sample B in geometries aligned with respect to VO_2_ 40–2_M_ or 002_R_ diffraction peak. The profiles are offset for clarity. Inset shows a schematic of unit cells of R phase VO_2_ and the incident and reflected x-ray.

The GST film was deposited by a rf magnetron sputtering method in an argon (Ar) atmosphere of 0.5 Pa with an output power of 100 W. Intentional substrate heating was not carried out. We prepared two types of samples with and without a SiN*_x_* buffer layer between GST and VO_2_ (see Section 3.2). The SiN*_x_* layer was grown by rf-sputtering a Si_3_N_4_ target with a power of 200 W under 0.5 Pa of Ar. Crystallization of the GST layer was achieved by postannealing the sample at 200 °C (with a heating speed of 20 °C min^−1^) for 2 min in Ar atmosphere or in air. In what follows, the step with the pristine VO_2_ layer before GST deposition is referred to as “VO”, the step with the as-deposited GST layer on VO_2_ as “AD”, and the step after the postannealing of the bilayered sample as “PA”. Figure 2 shows the X-ray diffraction (XRD) profiles of a GST/VO_2_ bilayered sample in steps AD and PA at RT in a geometry aligned with respect to VO_2_ 40–2_M_ or 002_R_ diffraction peak. The weak intensity of the Al_2_O_3_ substrate peaks is because the VO_2_ 100_M_ plane was slightly misoriented against the (1–102) plane of the substrate. The absence of GST peaks before annealing proved the amorphous nature of the as-deposited GST layer, while the diffraction peaks corresponding to NaCl-type GST that appeared after annealing indicated the success of the crystallization process. The *c*_R_ axis is always supposed to lie in plane in VO_2_ films grown on C-plane Al_2_O_3_, whereas one of three geometries allows the *c*_R_ axis to lie in plane on R-plane Al_2_O_3_. Table 1 shows the thickness of each layer in the four samples (A–D) reported in the present article.

### 2.2. Characterization

To investigate *T*_tr_ of VO_2_ at each step, we performed temperature-controlled measurements of optical reflection intensity and electrical resistance.

Optical reflection intensity was measured in an Ar atmosphere of 1 atm using a heating/cooling stage (HFS-91, Linkam, Tadworth, UK) installed in an optical microscope. White light from a halogen lamp was shone on the sample surface through a half mirror, and the reflected light was detected by a laser power meter (Vega, Ophir, Jerusalem, Israel). The sample temperature was swept at a rate of either 5 °C min^−1^ or 3 °C min^−1^. In order to perform the measurement to temperatures below RT, liquid nitrogen-cooled air was supplied in this stage. It is known that the temperature dependence of the optical properties of GST is negligible in the whole temperature range of the measurements (−20 °C minimum and 120 °C maximum) [29].

It is possible to evaluate the resistance of the VO_2_ layer only when the insulating buffer layer is inserted between GST and VO_2_ layers, since the resistivity of the crystalline GST is comparable with that of VO_2_. A 10 nm thick SiN*_x_* layer was employed as the insulating layer. The resistance as a function of temperature (*R–T*) of the VO_2_ layer in GST/SiN*_x_*/VO_2_ multilayered samples was measured with a two-probe scheme using tungsten–carbide (WC) probes. Both edges of the VO_2_ layer were covered during deposition of SiN*_x_* and GST layers to make these areas accessible by the probes. The resistance was measured by a multimeter (2000, Keithley, Solon, OH, USA), while the sample temperature was swept with a homemade temperature control stage, in which the power for a Peltier device was controlled by a computer.

## 3. Results

### 3.1. Optical Reflection

**Figure 3:**
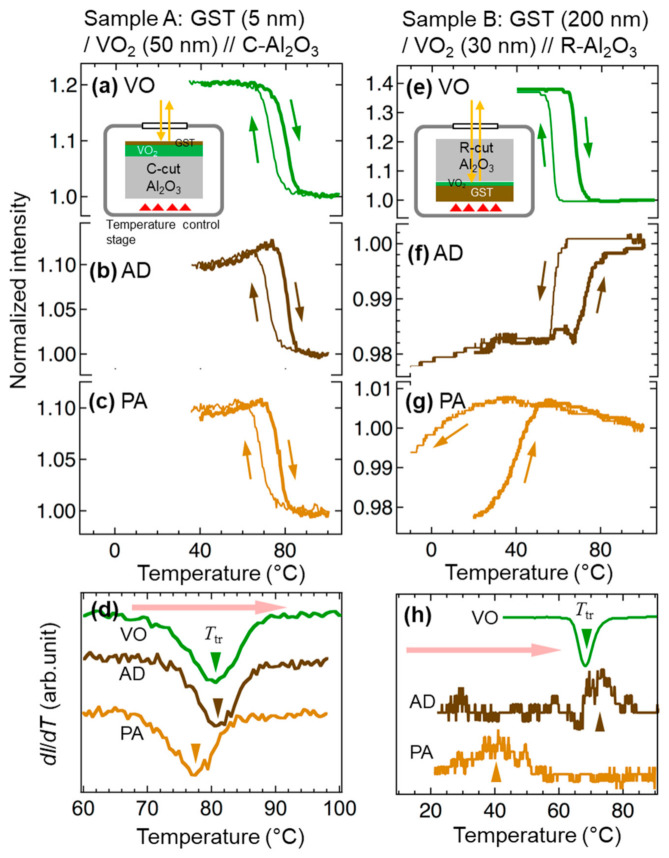
Temperature dependence of optical reflection intensity from Sample A (**a**–**c**) and Sample B (**e**–**g**) in steps VO (**a**,**e**), AD (**b**,**f**), and PA (**c**,**g**) during heating (thick lines) and cooling (thin lines) runs. The intensity is normalized at 100°C. The incident light was shone upon the film side (Sample A) or from substrate side (Sample B), as illustrated in insets of (**a**) and (**e**). (**d**,**h**) Temperature-differential profiles of reflection intensity (*dI/dT*) in the heating runs for Samples A and B, respectively, at the three steps. The triangle symbols indicate the peak/valley position.

For Sample A with the ultrathin GST layer of 5 nm thick, we observed the reflection from the film side. Figure 3a–c show the temperature dependence of the optical reflection intensity of Sample A in steps VO, AD, and PA, respectively. The intensity is normalized with the values at 100 °C. Significant evolution of ~10% in reflectance was detected even through the GST layer (Figure 3b,c). The sharp change of the reflectance was attributed to the phase transition of VO_2_. Figure 3d shows the temperature differential profiles of the reflected light intensity (*dI/dT*) in the heating runs in the three steps. A slight lowering of *T*_tr_, i.e., by about 4 °C, was revealed in step PA with respect to step AD. It is striking that the stress-induced modulation of *T*_tr_ in VO_2_ was realized even when the VO_2_ layer was 10 times thicker (50 nm) than the GST layer (5 nm). For optical switch applications, combinations of a thin GST layer and a thick VO_2_ layer may be of use.

Figure 3e–g show the results of similar observations of Sample B. For this sample, the reflection measurements were carried out in a substrate-side incident configuration, since the opaque nature of 200 nm thick GST prevented taking measurements on the film-side. The reflection intensity on the low-temperature side was stronger than that on the high-temperature side in step VO (Figure 3e), whereas the temperature dependence was inverted in steps AD and PA (Figure 3f,g). To confirm if such a difference in temperature dependence was reasonable, we calculated the reflectivity at a wavelength of 700 nm by using analytical solutions of electromagnetic waves [44] that propagate in multilayered structures, based on the dielectric functions of SiO_2_ and VO_2_ taken from the literature [45,46], and that of GST measured by us. For a sample with a pristine VO_2_ layer, modeled with a SiO_2_ (semi-infinite thickness)/VO_2_ (30 nm thick)/vacuum (semi-infinite thickness) multilayered structure, the electromagnetic calculation reproduced higher reflectivity when the VO_2_ was in the M phase compared to the R phase. With a GST layer [SiO_2_ (semi-infinite)/VO_2_ (30 nm)/GST (200 nm)/vacuum (semi-infinite) structure], on the other hand, the calculations revealed lower reflectivity in case of the M phase VO_2_ compared to the R phase, regardless of phases in GST. These simulations were consistent with the experimental results, supporting the hypothesis that both types of abrupt changes in the reflection intensity observed in Samples A and B were caused by the temperature-induced M–R or R–M phase transition.

Figure 3h shows the *dI*/*dT* profiles in the heating runs in the three steps. Either the valley or the peak in each curve is supposed to correspond to the phase transition. The center temperature of transition during heating runs existed at 68 and 72 °C in steps VO and AD, respectively, suggesting that deposition of the GST layer did not drastically affect *T*_tr_. In contrast, the postannealing process obviously lowered the *T*_tr_. The transition in step PA took place in a range of 25–50 °C with the center temperature being ~41 °C, suggesting a lowering of *T*_tr_ by approximately 30 °C caused by annealing. The *T*_tr_ changed more drastically in Sample B compared with Sample A; this can be understood by supposing that the smaller the thickness ratio between VO_2_ and GST layers, the more stress the VO_2_ layer will suffer from the shrinkage of the GST layer.

### 3.2. Electrical Resistance

Here, one may wonder if the lowering of *T*_tr_ reported above was an interdiffusion effect, which may have occurred during the postannealing at 200 °C. The possibility of the Ge-doping effect was rejected, since it is known to increase the *T*_tr_ [47]. Still, a Sb- or Te-doping effect cannot be excluded at this moment. The migration of oxygen ions from VO_2_ towards GST could cause the reduction of VO_2_, which could be another factor to decrease its *T*_tr_ [48,49]. Proof of the strain effect on the decrease of *T*_tr_ requires a way to prevent interdiffusion. In addition, the GST and VO_2_ layers should be insulated from each other when one evaluates the electrical conductivity of the VO_2_ layer in step PA, since the conductivity of the crystallized GST is comparable with that of R-phase VO_2_. To distinguish the GST crystallization effect from the interdiffusion effect, and to achieve *R–T* measurements of solely the VO_2_ layer under GST, we prepared samples with a SiN*_x_* buffer layer, which was intended to play two roles, i.e., as an interdiffusion barrier and an insulator.

**Figure 4:**
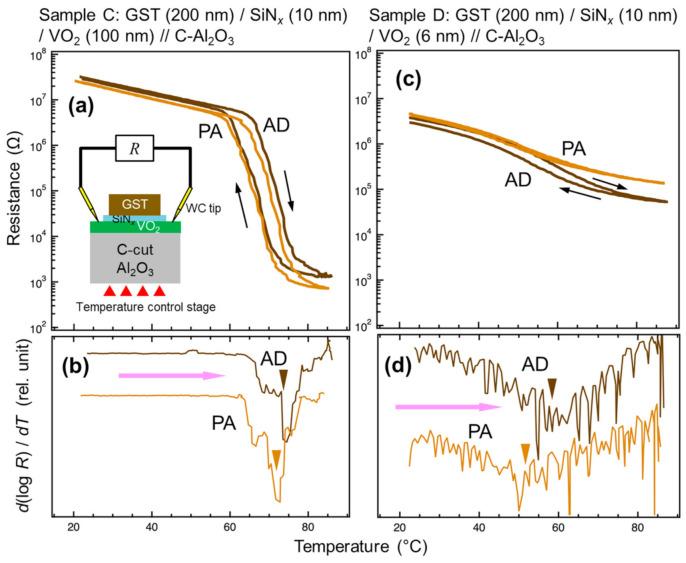
(**a**–**d**) Temperature dependence of electrical resistance (**a**,**c**) and temperature-differential profiles of the logarithm of the resistance [*d*(log *R*)/*dT*] in the heating runs (**b**,**d**) for Sample C (**a**,**b**) and Sample D (**c**,**d**) at AD and PA steps. Inset of (**a**) schematically shows the measurement configuration. The triangle symbols indicate the valley position.

Figure 4a shows the *R–T* curves of Sample C with a thick VO_2_ layer (100 nm) in steps AD and PA. In both cases, the phase transition was clearly observed through significant resistance change, i.e., four orders of magnitude. The comparable resistance values in the M phase in both steps implied that the SiN*_x_* layer functioned as the insulator, preventing the current flow in the crystalline GST layer. Figure 4b shows temperature differential profiles of logarithm of the resistance of Sample C in the heating runs. One can see a shift of the valley between steps AD and PA, which suggests the decrease of the *T*_tr_ caused by the postannealing, even with a configuration where the interdiffusion effect was excluded. It was assumed that this behavior was the result of shrinkage of the *c*_R_ axis caused by in-plane compressive strain due to the crystallization of GST. The fitting to Pseudo-Voigt functions of the differential curves indicated a *T*_tr_ of ~74 and ~72 °C in steps AD and PA, respectively.

**Figure 5:**
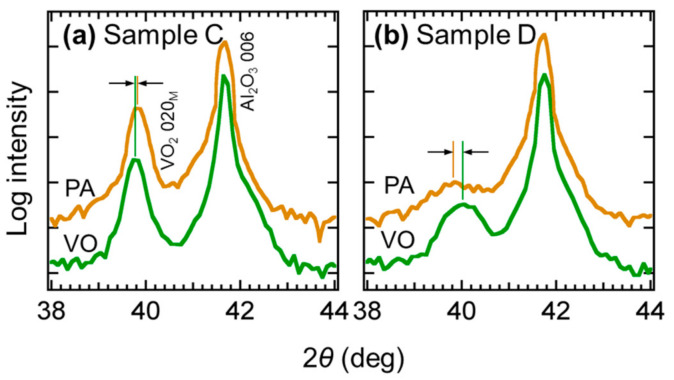
XRD 2*θ − ω* scan profiles of Samples C (**a**) and D (**b**) in steps VO and PA.

We performed a similar experiment for another sample with a thinner VO_2_ layer (6 nm, Sample D). The *R–T* curves of the sample showed a resistance change of more than one order of magnitude, suggesting the existence of a weak phase transition (Figure 4c). The fitting of the temperature differential profiles revealed *T*_tr_ of ~58 and ~52 °C in steps AD and PA, respectively, suggesting a lowering of *T*_tr_ for about 6 °C during the crystallization of GST (Figure 4d). Thinner than the VO_2_ layer in sample C, the VO_2_ layer in this sample was probably more severely affected by the stress from GST. To qualitatively observe the difference of the degree of stress, we performed XRD 2*θ − ω* scans of Samples C and D in steps VO and PA (Figure 5). In Sample D, a shift of the VO_2_ 020_M_ peak to a lower angle occurred when the ultrathin VO_2_ layer was capped with crystalline GST. The elongation of the out-of-plane lattice suggested the shrinkage of the in-plane lattice and *c*_R_ axis, which is in agreement with our understanding. In contrast, no significant shift was observed in Sample C with a thick VO_2_ layer, which was consistent with the less drastic shift of its *T*_tr_.

## 4. Discussion

The transition temperatures obtained from all the experiments in this study are summarized in Table 1. It is easy to predict that the lower the thickness ratio between VO_2_ and GST layers, the larger the stress that VO_2_ suffers from GST when the GST is crystallized. The tendency found in the results, i.e., a more significant drop of *T*_tr_ in the sample with smaller VO_2_/GST thickness ratio, supports the hypothesis that the modification of *T*_tr_ was induced by the stress which occurred during the crystallization of GST. In the present study, mechanical strain was introduced into the VO_2_ films in situ, unlike the previous films on TiO_2_ (001) with static strain. On the other hand, studies on piezo-induced in situ *T*_tr_ modification of VO_2_ resulted in reductions of only 1.35 °C [23] or 6 °C [24]. The strain that would be induced through the volume change at an amorphous–crystalline phase change (2.3%) could be significantly superior to that induced through the piezoelectric effect (± 0.2%) [50].

Nevertheless, the shift of *T*_tr_ for ~6 °C, observed in *R–T* measurements (Sample D), was not as huge as that observed in reflectance measurements, ~30 °C (Sample B). One reason for this may be that the optical reflectance is sensitive in detecting the property change at the GST/VO_2_ interface, whereas the electrical resistance contains the property of the VO_2_ film for the whole thickness. The VO_2_ lattice at the film/substrate interface was probably pinned by the substrate lattice, even when that at the GST/VO_2_ interface was shrunk due to stress from GST. The pinning effect could be pronounced when the VO_2_ layer is thin. Optimization of the thicknesses of the GST, VO_2_, and buffer layers would be required. Another reason for this, in particular regarding Samples C and D, could be that the SiN*_x_* layer may have absorbed a large part of the stress from the GST layer and weakened the deformation of the VO_2_. ZnS–SiO_2_, an insulating material commonly used in optical disks, is supposed to be softer than SiN*_x_*, and could be useful for improving the transport efficiency of the stress. The change of *T*_tr_ for ~30 °C in the present results may not be large enough for device applications, and therefore, transportation of the 2.3% strain from GST to VO_2_ with a higher efficiency would be sought after.

More importantly, the application to optical devices such as memories and switches would require an amorphization process of the crystalline GST layer. Amorphization is currently performed in commercial optical disks by rapid local heating of the material, which should be in the order of μm^2^ in area and tens of ns in duration [51]. Such a process will be examined in our future research.

## 5. Conclusions

In GST/VO_2_ bilayered films prepared on Al_2_O_3_ substrates, the phase transition properties of the VO_2_ layers were compared between the steps before and after crystallization of the GST layer. It was shown that the crystallization of the GST layer lowers the phase transition temperature of the VO_2_ layer, either with or without a SiN*_x_* buffer layer. The nonvolatile modification of *T*_tr_ was probably induced through strain in the VO_2_ layer, which originated in the volume shrinkage of the GST layer at its amorphous–crystalline phase change. The shift of *T*_tr_ caused by the crystallization of the GST layer was by approximately 30 °C for a GST/VO_2_ bilayered sample with thicknesses of 200/30 nm. Once an amorphization process has been established, the presently-described devices will possibly be proved to function as reversible, nonvolatile resistance change memory or optical switching devices with operation temperature ranges of several tens of degrees Celsius. The operation mechanisms of the present samples would represent new guidelines in the strain engineering field, and would greatly broaden the possibilities of strain-driven devices.

## Figures and Tables

**Table 1:** Thickness of each layer, measurements performed at each step, and the results of *T*_tr_ in heating runs from each measurement in four samples.

Sample	VO_2_ Thickness (nm)	GST Thickness (nm)	SiN*_x_* Layer	Substrate	AD		PA	
					Meas.	*T*_tr_ (°C)	Meas.	*T*_tr_ (°C)
A	50	5	No	C-cut Al_2_O_3_	Refl.	81	Refl.	77
B	30	200	No	R-cut Al_2_O_3_	Refl.	72	Refl.	41
C	100	200	Yes	C-cut Al_2_O_3_	*R–T*	74	*R–T*	72
D	6	200	Yes	C-cut Al_2_O_3_	*R–T*	58	*R–T*	52

Meas. = measurement. Refl. = optical reflection intensity.
